# Evaluation of the Potential Diagnostic Utility of the Determination of Selected Caspases—Markers Involved in the Regulation of Apoptosis—In Patients with Ovarian Cancer

**DOI:** 10.3390/diagnostics11040704

**Published:** 2021-04-14

**Authors:** Aleksandra Mielczarek-Palacz, Sylwia Jasińska, Anna Strzelec

**Affiliations:** Department of Immunology and Serology, Faculty of Pharmaceutical Sciences in Sosnowiec, Medical University of Silesia, 40-055 Katowice, Poland; sylwia.j.1995@wp.pl (S.J.); anna.agata.strzelec@gmail.com (A.S.)

**Keywords:** caspase-3, caspase-8, caspase-9, ovarian cancer, apoptosis

## Abstract

Ovarian cancer remains a major diagnostic and therapeutic problem in modern gynecological oncology. For this reason, research which focuses on the search for new diagnostic markers and the assessment of their possible usefulness in clinical practice is still being conducted. The aim of this study was to evaluate serum levels of caspase-3, caspase-8, and caspase-9 in women with ovarian cancer. Patients with ovarian serous cystadenoma (*Cystadenoma serosum*) and papillary serous cystadenocarcinoma (*Cystadenocarcinoma papillare serosum IIIC*) were included in the study, as well as healthy women who constituted the control group. The results of the study revealed a statistically significantly decreased mean serum levels of caspase-3, caspase-8, and caspase-9 in women with ovarian cancer as compared to the control group (*p* ˂ 0.001), which indicates the involvement of the studied parameters in immune system disturbances occurring in the process of apoptosis by the extrinsic and intrinsic pathway and may be one of the mechanisms of immunosuppression accompanying these tumors. Determination of serum levels of examined caspases and CA 125 antigen in women with ovarian cancer in combination with other markers may prove useful in the future in the diagnosis of ovarian cancer, but this requires further studies.

## 1. Introduction

World epidemiological reports indicate a steady increase in the trend of coming down with the “silent killer”—ovarian cancer. More than 184,000 women with ovarian cancer die worldwide each year [1], as the cancer in more than 70% of affected women is still detected at clinical stage III or IV, which is associated with poor prognosis and low five-year survival rates [2]. Detection of the cancer in question at an early stage significantly increases the prognosis, with a five-year survival rate of 80–90% [3]. Ovarian cancer, therefore, remains a serious diagnostic and therapeutic problem in modern gynecological oncology. Considering that, research that focuses on the search for new prognostic and diagnostic markers and the evaluation of their possible usefulness in clinical practice is still being conducted [4,5,6].

Disturbed immune response directed against tumor cells, especially inhibition of apoptosis, plays a significant role in the pathogenesis of ovarian cancer [7]. This process may be mediated by several signaling pathways. The best-known pathways are the extrinsic and intrinsic pathways, involving characteristic initiator caspases [8,9]. In the case of the external apoptosis pathway, it is caspase-8, which is the active form of procaspase-8. It is activated by binding of the appropriate ligand through the death receptor, leading to the formation of the DISC (*Death-Inducing Signaling Complex*) complex and, in a further step, caspase-8, which initiates the cascade of effector caspases, resulting in the death of the target cell [10]. Mitochondrial damage, leading to disruption of cell homeostasis, triggers an intrinsic apoptotic pathway in which caspase-9 is the main initiator caspase and its inactive form, procaspase-9, combines with cytochrome c and Apaf-1 to form a cytosolic complex, the apoptosome, responsible for its activation. Caspase-9 activates a cascade of effector caspases, ultimately leading to the death of the target cell [11]. In the execution phase of apoptosis, caspase-3, considered as the most important of the effector caspases, plays a key role [8]. It is activated when the signaling pathway is triggered by one of the initiator caspases. Caspase-3, due to its enzymatic functions, is able to degrade cellular components, which leads to morphological changes and contributes to the death of the target cell [12].

Previous studies indicate the involvement of caspases in the normal functioning of the organism, and changes in their activity participate in the pathogenesis of many diseases which may be caused by both decreased and increased activity of these proteases [13]. In the case of cancer cells, abnormal expression of caspases, leading to impaired apoptosis and consequently promoting further tumor growth, may also contribute to treatment resistance [7,9]. Therefore, monitoring the course of apoptosis with the participation of soluble markers of this process—caspases—may find possible application in the development of new diagnostic and therapeutic strategies in cancer, including ovarian cancer [13]. The aim of this study was to analyze the soluble markers monitoring the apoptosis process by assessing the levels of caspase-3, caspase-8, and caspase-9 in the serum of women with ovarian cancer.

## 2. Material and Methods

The study involved 36 patients of the Municipal Hospital No. 2 in Ruda Śląska, Clinical Department of Gynaecology and Obstetrics, Department of Women’s Health, Medical University of Silesia in Katowice, aged 37 to 78 years (mean age: 58.1 years ± 10.9 years) with a diagnosis of ovarian tumors. In this group, 16 women were diagnosed with benign ovarian tumors, ovarian serous cystadenoma (*Cystadenoma serosum*), and 20 women with papillary serous cystadenocarcinoma (*Cystadenocarcinoma papillare serosum IIIC*). The clinical diagnosis of the tumor was confirmed by histopathological examination, while the stage of ovarian cancer was determined according to the FIGO *(International Federation of Gynecology and Obstetrics)* classification. The control group consisted of 14 healthy women aged 20 to 62 years (mean age: 43.5 years ± 13.7 years) in whom no pathological changes within the reproductive organs were found after prophylactic examinations. The material studied was venous blood serum collected “for clotting” from the elbow vein in the morning. All qualified women gave written consent to conduct the study, which was performed with the consent of the Bioethics Committee of the Medical University of Silesia in Katowice. The concentration of caspase-3, caspase-8, and caspase-9 in serum, was determined by immunoenzymatic method ELISA using Human Caspase-3 ELISA Kit, Human Caspase-8 ELISA Kit, and Human Caspase-9 ELISA Kit from Bender MedSystems (Vienna, Austria). The sensitivities of the tests were, respectively: 0.12 ng/mL, 0.4 ng/mL, and ˂0.4 ng/mL. The concentration of cancer antigen CA 125 was determined by a microparticle enzyme immunoassay (MEIA) using Tumor Markers CA-125TM MEIA Kits from Abbott Diagnostics with an assay sensitivity of 2 U/mL.

The obtained results were statistically analyzed using Statistica 13.3 and PQStat 1.6.8 software. The normality of distribution of the studied variables was checked using the Shapiro–Wilk test. For caspase-3, caspase-8, and caspase-9, arithmetic means and standard deviations were calculated with Student’s *t*-test, and for CA 125 antigen, the median and quartile range were determined with Mann–Whitney *U* test. Pearson’s test was used to assess the relationship between the studied parameters and the decimal logarithm from the CA 125 antigen concentration. The assessment of the statistical significance of the difference between the area under the Receiver Operating Characteristic (ROC) curve of the studied caspases and the area under the ROC curve of the CA 125 antigen was performed using DeLong’s test; the level of *p* ˂ 0.05 was considered as statistically significant.

## 3. Results

Serum levels of caspase-3, caspase-8, caspase-9, and CA 125 antigen were determined in healthy women belonging to the control group and women with ovarian cancer.

**Figure 1:**
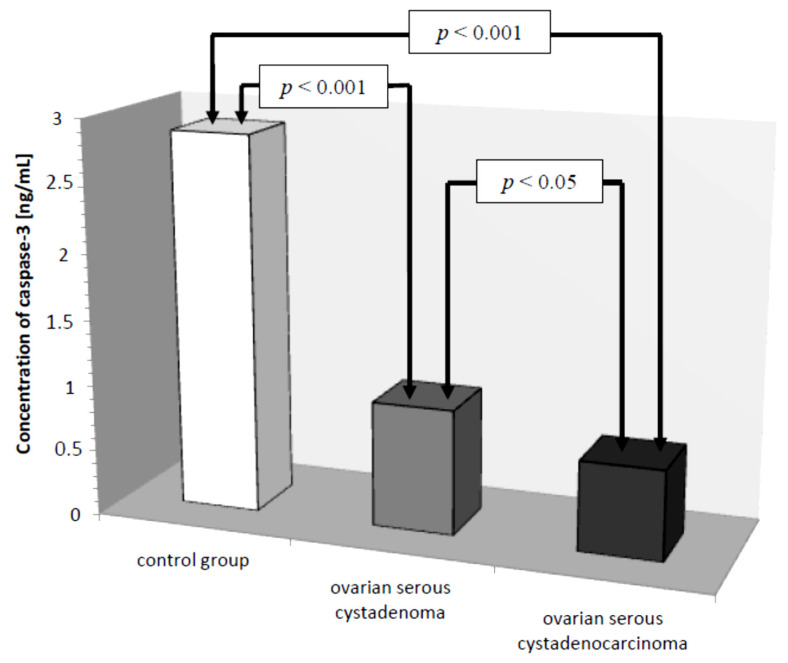
Mean serum caspase-3 levels in women with ovarian serous cystadenoma, ovarian serous cystadenocarcinoma, and control group.

In the serum of healthy women, the lowest determined concentration of caspase-3 was 1.26 ng/mL, the highest 4.70 ng/mL, with the mean value of 2.87 ± 1.06 ng/mL. In the study group, the concentration of the determined parameter ranged from 0.16 ng/mL to 1.72 ng/mL, with a mean value of 0.79 ± 0.42 ng/mL, and was statistically significantly lower compared to the control group (*p* ˂ 0.001). Next, the serum levels of caspase-3 in women with ovarian serous cystadenoma and papillary serous cystadenocarcinoma were analysed. The analysis showed a statistically significantly lower concentration of the examined parameter in the serum of women with papillary serous cystadenocarcinoma compared to its concentration in women with ovarian serous cystadenoma (*p* ˂ 0.05). In addition, there were statistically significantly lower levels of caspase-3, both in the serum of women with ovarian serous cystadenoma and papillary serous cystadenocarcinoma, compared to the serum levels of women in the control group (*p* ˂ 0.001), as illustrated in Figure 1.

**Figure 2:**
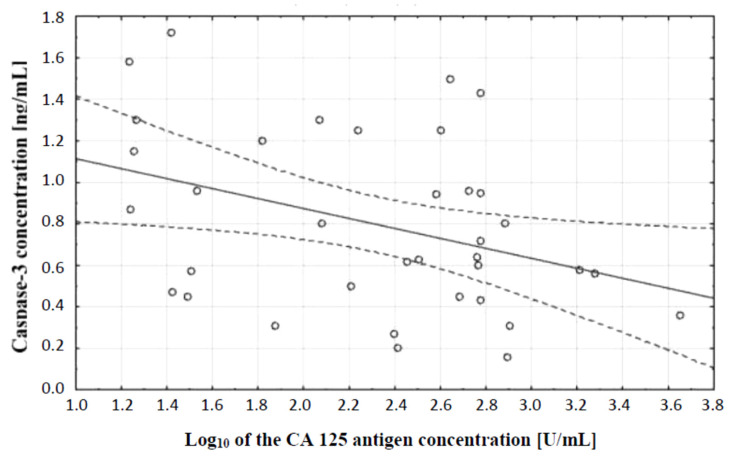
Linear regression curve showing the relationship between caspase-3 concentration and the decimal logarithm of CA 125 antigen concentration in the blood serum of women with ovarian cancer.

In the next stage of statistical analysis, a correlation test, which showed the existence of a relationship between the concentration of caspase-3 and the concentration of CA 125 antigen in the serum of the examined women, was performed. There was a negative (*r* = −0.38) statistically significant (*p* ˂ 0.05) correlation between the variables, which is illustrated in Figure 2.

**Figure 3:**
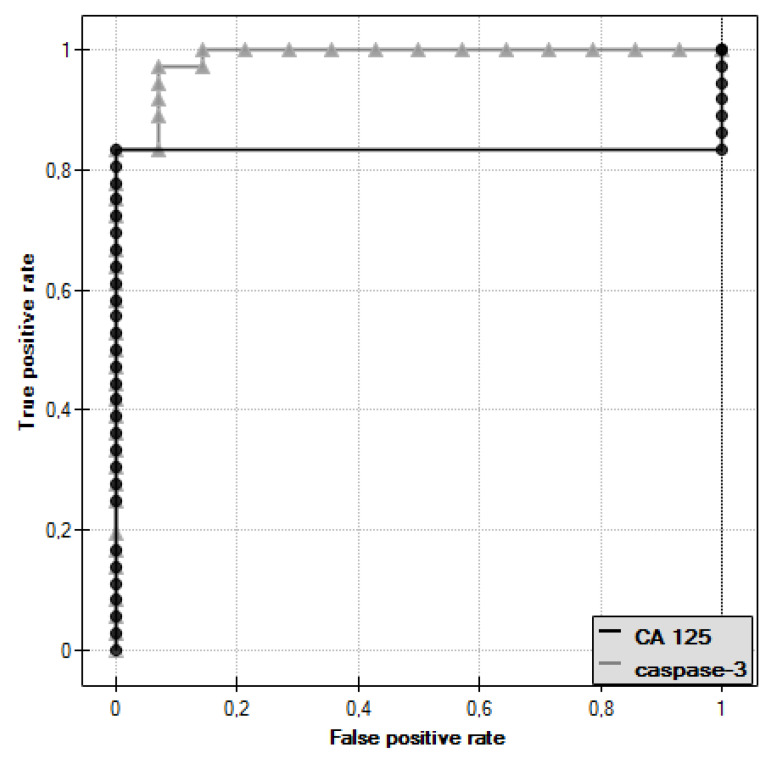
Receiver Operating Characteristic (ROC) curve of caspase-3 and ROC curve of CA 125 antigen.

In the course of further analysis, Receiver Operating Characteristic (ROC) curves for caspase-3 and CA 125 antigen were plotted, and the values of the Area Under Curve (AUC) were determined—for caspase-3: AUC = 0.986, and for CA 125: AUC = 0.833 (Figure 3).

The diagnostic value of caspase-3 and CA 125 antigen determination was evaluated by determining the sensitivity and specificity for these parameters, as shown in Table 1. For CA 125 antigen, the cut-off point was 35 U/mL, while for caspase-3, the cut-off point determined was 1.72 ng/mL.

**Figure 4:**
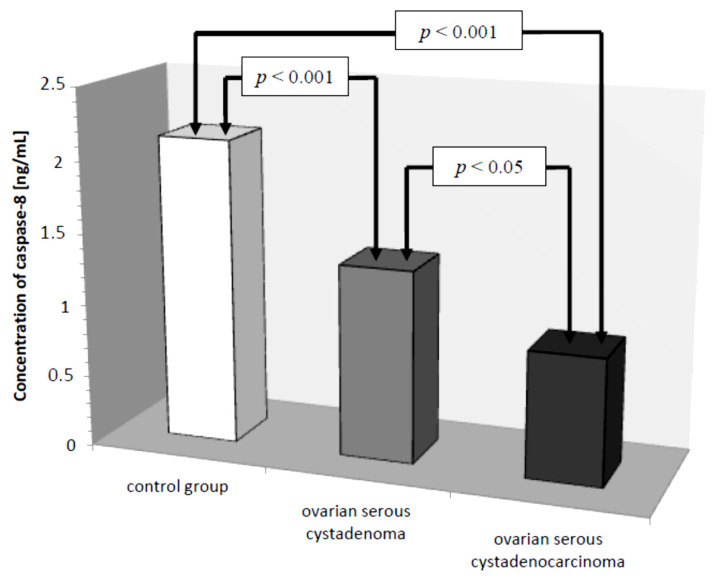
Mean serum caspase-8 levels in women with ovarian serous cystadenoma, ovarian serous cystadenocarcinoma, and control group.

Subsequently, caspase-8 serum levels were analyzed in all the women studied. In the control group, the lowest concentration of caspase-8 was 1.50 ng/mL and the highest was 3.20 ng/mL, the mean value was 2.13 ± 0.49 ng/mL. In the group of women with ovarian cancer, the concentration of the determined parameter ranged from 0.25 ng/mL to 2.50 ng/mL, with a mean value of 1.08 ± 0.61 ng/mL, which was statistically significantly lower compared to the control group (*p* ˂ 0.001). Further analysis revealed a statistically significantly lower concentration of the test parameter in the serum of women with serous carcinoma compared to that of women with ovarian serous adenocarcinoma (*p* ˂ 0.05) and a statistically significantly lower serum concentration of caspase-8 in both women with ovarian serous cystadenoma and papillary serous cystadenocarcinoma compared to that in women in the control group (*p* ˂ 0.001), as illustrated in Figure 4.

**Figure 5:**
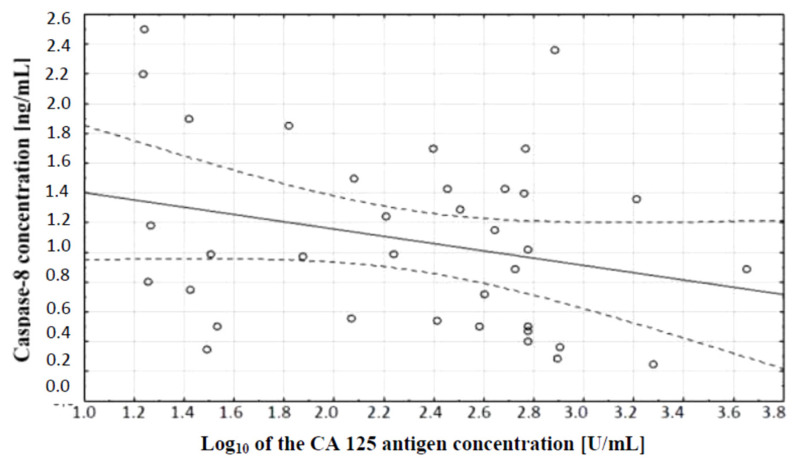
Linear regression curve showing the relationship between caspase-8 concentration and the decimal logarithm of CA 125 antigen concentration in the blood serum of women with ovarian cancer.

The comparison of caspase-8 and CA 125 antigen concentrations in serum of women with ovarian cancer and healthy women was also made. A correlation test, which showed the existence of a relationship between caspase-8 concentration and serum CA 125 antigen concentration in the examined women, was performed. A negative (*r* = −0.27) statistically insignificant (*p* ˃ 0.05) correlation between the variables was demonstrated, which is illustrated in Figure 5.

**Figure 6:**
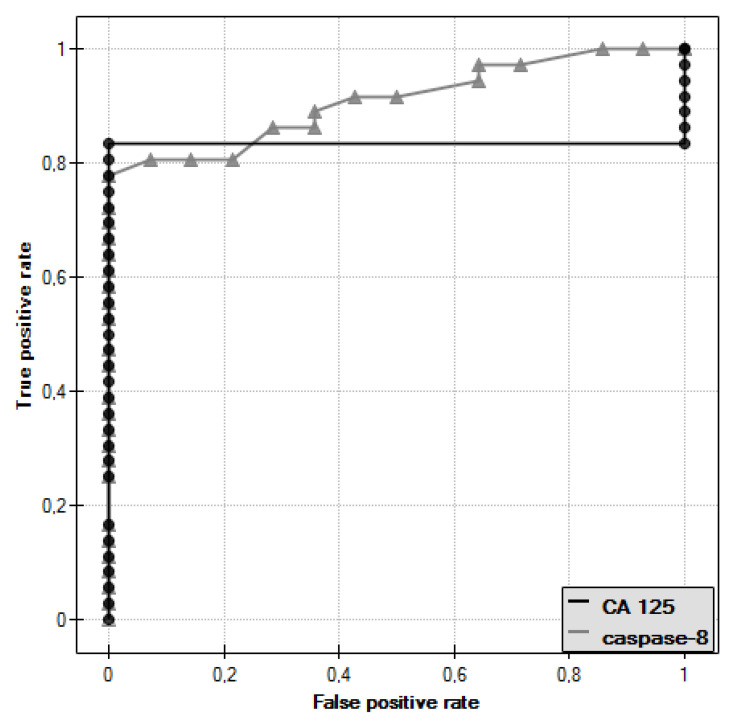
ROC curve of caspase-8 and ROC curve of CA 125 antigen.

In the course of further analysis, ROC curves for caspase-8 and CA 125 antigen were plotted and the values of the areas under them–AUC–were determined, for caspase-8: AUC = 0.909, and for CA 125: AUC = 0.833 (Figure 6).

The diagnostic value of the determination of caspase-8 and CA 125 antigen was evaluated by determining the sensitivity and specificity, as shown in Table 2. For CA 125 antigen, the cut-off point was taken as 35 U/mL, while for caspase-8, the cut-off point determined was 1.50 ng/mL.

Another parameter analyzed was caspase-9, whose concentration in the control group ranged from 15.20 ng/mL to 32.40 ng/mL with a mean value of 23.01 ± 4.70 ng/mL. In women with ovarian cancer, the mean caspase-9 concentration was 4.02 ± 1.88 ng/mL ranging from 1.40 ng/mL to 8.30 ng/mL and was significantly reduced compared to the control group (*p* ˂ 0.001). Next, the mean serum caspase-9 levels in women were examined, taking into account the type of ovarian cancer diagnosed.

**Figure 7:**
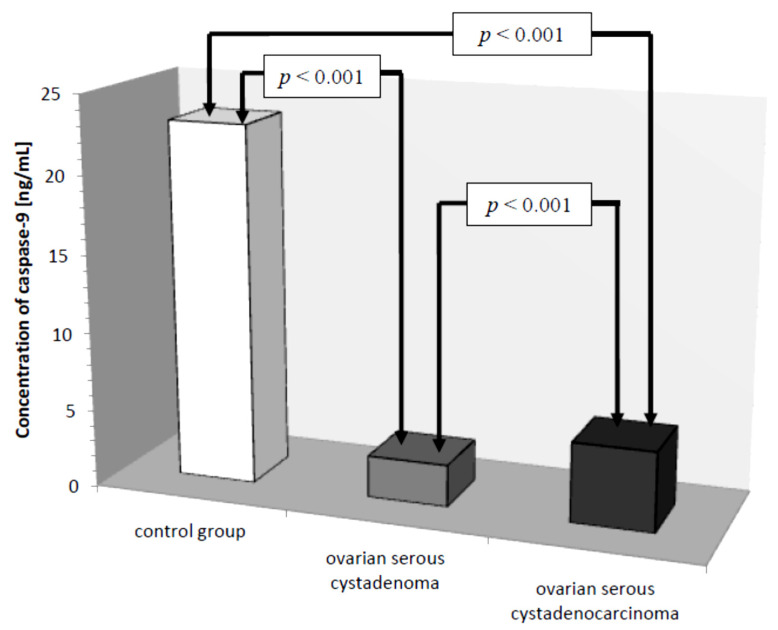
Mean serum caspase-9 levels in women with ovarian serous cystadenoma, ovarian serous cystadenocarcinoma, and control group.

There was a statistically significant difference between serum caspase-9 levels in women with ovarian serous cystadenoma and papillary serous cystadenocarcinoma (*p* ˂ 0.001) and there were statistically significantly lower serum levels of the studied parameters in women with these tumors compared to serum levels in healthy women (*p* ˂ 0.001), as shown in Figure 7.

**Figure 8:**
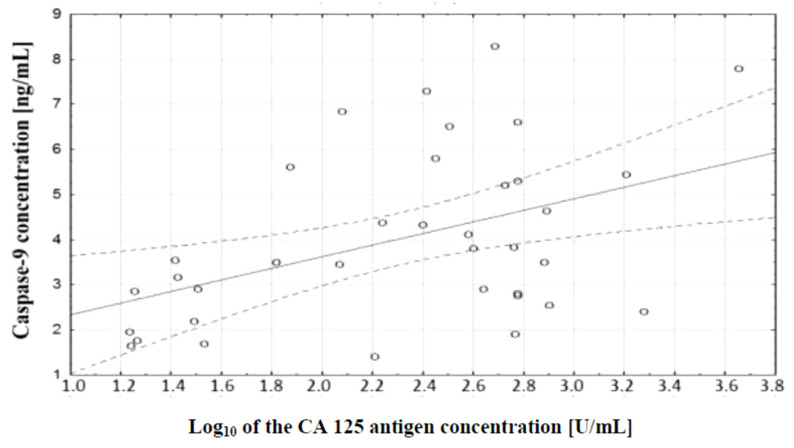
Linear regression curve showing the relationship between caspase-9 concentration and the decimal logarithm of CA 125 antigen concentration in the blood serum of women with ovarian cancer.

In the course of further analysis, a correlation test, which demonstrated the existence of a relationship between caspase-9 concentration and serum CA 125 antigen concentration in the examined women, was performed. There was a positive (*r* = 0.48) statistically significant (*p* ˂ 0.01) correlation between the variables. This relationship is illustrated in Figure 8.

**Figure 9:**
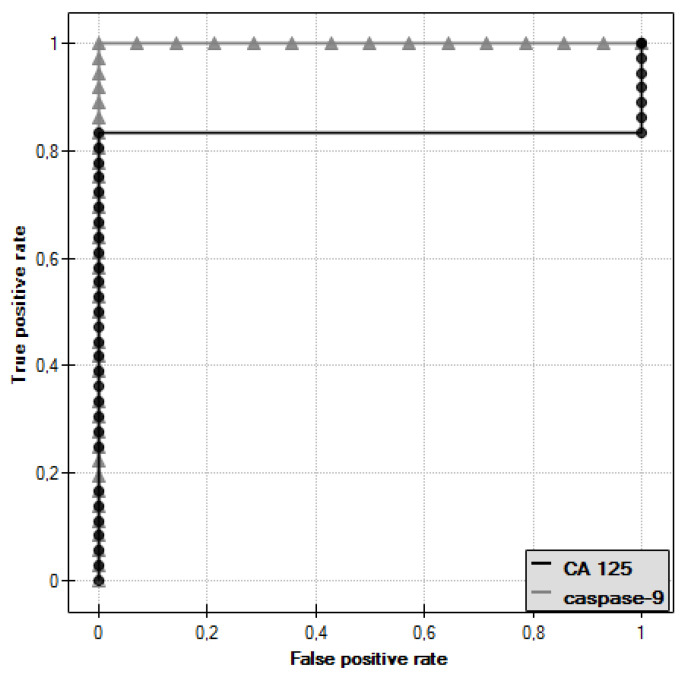
ROC curve of caspase-9 and ROC curve of CA 125 antigen.

ROC curves were plotted for caspase-9 and CA 125 antigen and the values of the areas under them–AUC–were determined, which were, respectively, 1 for caspase-9 and 0.833 for CA 125 (Figure 9).

In the last stage of analysis, the diagnostic value of caspase-9 and CA 125 antigen determination was evaluated by determining the sensitivity and specificity, as shown in Table 3. For CA 125 antigen, the cut-off point was taken as 35 U/mL, while for caspase-9, the cut-off point determined was 8.30 ng/mL.

## 4. Discussion

The high mortality rate of ovarian cancer is associated with the diagnosis of cancer at an advanced clinical stage, which may be due to the latent development of the disease and the lack of effective early detection methods that could be helpful in the diagnosis of these tumors [14,15].

There is ample evidence that proteins critical for the transmission of programmed death signals are involved in the formation and development of ovarian cancers [16,17]. It also appears that proteins that inhibit apoptosis may act as oncogenes and potentially an increase in their expression will promote tumor formation. However, this process is extremely complex and disruption of apoptosis may be one of its more important steps [18].

Previous studies indicate that some cancer cells may show sensitivity to factors inducing the extrinsic and intrinsic pathway of apoptosis, therefore, it seems appropriate to study markers of this process, including cysteine proteases, called caspases. Previous studies have shown a link between disruption of their expression and cancer pathogenesis [19,20]. Numerous studies are being conducted to assess the role of selected caspases in cancer patients and their parameter determinations in terms of clinical utility.

Therefore, the aim of the present study was to evaluate serum levels of caspase-3, caspase-8, and caspase-9 in women with ovarian cancer. The study included patients with ovarian serous cystadenoma (*Cystadenoma serosum*) and papillary serous cystadenocarcinoma (*Cystadenocarcinoma papillare serosum IIIC*) and healthy women who constituted the control group. Statistical analysis of the obtained results revealed significantly decreased mean serum levels of caspase-3, caspase-8, and caspase-9 in women with ovarian cancer compared to the control group (*p* ˂ 0.001), which indicates the involvement of the studied parameters in immune system disturbances occurring in the process of apoptosis by extrinsic and intrinsic pathways in patients with ovarian cancer.

The analysis of serum caspase-3 concentration provided interesting observations. It showed a statistically significant difference between the concentration of this parameter in serum of women with ovarian serous cystadenoma and papillary serous cystadenocarcinoma, which indicates an intensification of disturbances occurring in the course of ovarian cancer. Decreased serum caspase-3 levels in women with diagnosed cancer may be related to decreased release of the enzyme from tumor cells due to disturbances in the executive phase of apoptosis, which may result from abnormal expression of initiator caspases that activate caspase-3 or a direct effect on this enzyme. Moreover, the analysis showed that there was a negative statistically significant correlation (*r* = −0.38, *p* ˂ 0.05) between the concentration of caspase-3 and the decimal logarithm of serum CA 125 antigen concentration in women from the study group, and the assessment of the diagnostic value of the tests showed that the determination of caspase-3 and serum CA 125 marker concentrations may prove useful in the diagnosis of ovarian cancer, but this requires further studies.

So far, there have been no studies on the evaluation of caspase-3 serum levels in women with ovarian cancer, but the expression of this enzyme in patients with ovarian cancer has been evaluated.

Such a study was conducted by Budiana et al. [12], who demonstrated reduced caspase-3 activity in 52.4% of cases while normal in 47.6% of patients with epithelial ovarian cancer. The study showed differences in caspase-3 expression depending on the degree of cell differentiation and stage of the disease. Patients with reduced activity of the enzyme showed a low degree of cell differentiation and advanced stage of ovarian cancer, as well as higher aggressiveness. Similar conclusions were reached by Duo and Tong [21], evaluating caspase-3 expression in women with primary ovarian cancer. They demonstrated a negative correlation between caspase-3 expression and the degree of cell differentiation and stage of the disease. Similarly, the study by Chen and Peng [22] showed a significantly lower expression of caspase-3 in tissues obtained from patients with ovarian cancer and borderline tumor of this organ compared to tissues obtained from patients with benign neoplasm and healthy individuals. Additionally, Hassan et al. [23], comparing the expression of caspase-3 in healthy subjects, in patients with benign ovarian tumor, and in women with ovarian cancer, showed a lower expression of the enzyme in the group of patients with benign and malignant tumor compared to healthy subjects. Moreover, caspase-3 activity in women with ovarian cancer was lower than in patients with benign tumors. Previous studies on the evaluation of caspase-3 activity in the prognosis of survival of women with ovarian cancer are divergent. According to Kleinberg et al. [24], low activity of the enzyme in tumor cells was associated with shorter survival. In contrast, the study by Espinosa et al. [25] showed a shorter survival of women in whom low caspase-3 activity was observed, and additional immunohistochemical tests performed showed that this enzyme was not present in tumor cells, but originated from tissue macrophages. Kim et al. [26] concluded from their study that low caspase-3 activity in women with ovarian cancer was associated with longer patient survival. Abnormal apoptosis allowing cells to undergo malignant transformation also occurs in patients with cervical cancer. Hu et al. [27], evaluating caspase-3 activity in women with this cancer, found high expression of this enzyme in 10.6% of patients and observed a negative correlation between caspase-3 expression and overall survival of patients with cervical cancer.

Analysis of caspase-3 expression was also performed in women with breast cancer [28]. The study showed the absence of activity of this enzyme in 75% of the affected women, and in the remaining patients, the expression was significantly reduced. However, Nakopoulou et al. [29], using an immunohistochemical assay to evaluate caspase-3 activity in women with invasive breast tumor, found high expression of the enzyme in 75.2% of patients. A similar study was conducted by Jha et al. [30] evaluating the expression of this enzyme in patients with benign and malignant breast cancer. Their study showed lower caspase-3 activity in women with breast cancer compared to patients with benign tumors.

Further analysis included evaluation of caspase-8 serum levels in women with ovarian cancer. Serum caspase-8 levels were found to be significantly lower in women with papillary serous cystadenocarcinoma (*p* ˂ 0.001) and ovarian serous cystadenoma (*p* ˂ 0.05) compared to serum levels of this parameter in healthy women. Decreased caspase-8 levels in the serum of women diagnosed with cancer may be related to reduced release of the enzyme from tumor cells due to disturbances in the extrinsic apoptotic pathway. A negative non-significant correlation was found between caspase-8 concentration and the decimal logarithm of serum CA 125 antigen concentration in women from the study group (*r* = −0.27). Furthermore, it was concluded that the assessment of serum caspase-8 and CA 125 marker levels may be useful in the diagnosis of ovarian cancer, but this requires further research.

There were no studies on the evaluation of caspase-8 serum levels in women with ovarian cancer, but the expression of this enzyme in patients with ovarian cancer was evaluated.

Such studies were conducted by Hassan et al. [23] who demonstrated lower caspase-8 expression in women with benign and malignant ovarian cancer compared to expression in healthy women. In addition, caspase-8 expression was lower in women with cancer compared to non-malignant cancer. A similar study was also conducted by Borhani et al. [31], who also showed lower caspase-8 expression in ovarian tissues collected from patients with cancer compared to expression in healthy controls.

Interesting observations are also provided by the study of Kim et al. [26], who showed that low caspase-8 activity in women with ovarian cancer was associated with shorter overall survival. In contrast, patients with higher enzyme activity had a longer survival period. Moreover, they found that impaired caspase-8 expression in ovarian cancer occurs as a result of mutations, deletions, or complete lack of expression of the enzyme. On the other hand, Kostova et al. [32], in their work, drew attention to the role of caspase-8 in the tumor microenvironment. Abnormal expression of this enzyme in ovarian cancer leads to an imbalance between apoptotic and non-apoptotic functions of caspase-8, not only in the primary tumor, but also in the surrounding environment. The presence or absence of caspase-8 determines the invasiveness and migration of tumor cells, and decreased expression of this enzyme in women with ovarian cancer is associated with increased aggressiveness and poor prognosis due to resistance to therapy.

Further analysis included the assessment of serum caspase-9 levels. There was a statistically significant difference between serum levels of this parameter in women with serous ovarian adenocarcinoma and serum levels in women with serous ovarian cancer (*p* ˂ 0.001). The values were statistically significantly lower compared to serum levels in healthy women (*p* ˂ 0.001), which may be related to reduced release of the enzyme from tumor cells due to disturbances in the intrinsic apoptosis pathway. The presence of a positive statistically significant correlation between caspase-9 concentration and the decimal logarithm of serum CA 125 antigen concentration in women from the study group was demonstrated (*r* = 0.48, *p* ˂ 0.01). Furthermore, it was concluded that assessment of serum caspase-9 and CA 125 marker concentrations may prove useful in the diagnosis of ovarian cancer, but this requires further research.

There have been no studies to date on the assessment of caspase-9 levels and the evaluation of the expression of this enzyme in patients with ovarian cancer and other gynecological cancers.

However, the expression of caspase-8 and caspase-9 was evaluated in patients with colorectal cancer. In normal tissue, caspase-9 was expressed along the entire length of the crypts, while caspase-8 was most strongly expressed in the surface epithelium of the intestinal lumen [33]. A significant decrease in caspase-9 activity and concomitant overexpression of caspase-8 was found in tissues obtained from patients with colorectal cancer compared to tissues obtained from healthy individuals. A correlation was also found between the expression of the enzymes and the degree of cell differentiation and the occurrence of metastases [34].

Similar conclusions were reached by Jäger et al. [35] finding low expression of caspase-9 in 46% of patients with colorectal cancer and normal or slightly elevated expression of caspase-8 in patients. Furthermore, assessment of the activity of these enzymes may be important in the prognosis of patient survival, as confirmed by Sträter et al. [33], who observed that low expression of both caspase-8 and caspase-9 was associated with shorter survival of patients with colorectal cancer.

## 5. Conclusions

Women with ovarian cancer have impaired signaling in the intrinsic and extrinsic pathways of apoptosis but also in the executive phase, which may be one of the mechanisms of immunosuppression associated with ovarian cancer.

The observed changes in serum concentrations of soluble markers monitoring the apoptosis process in diseased women, especially intensified in women with serous ovarian cancer, are related to tumor diagnosis and may prove useful possibly in the differential diagnosis of these tumors, which requires further studies.

Determination of analyzed caspases and CA 125 antigen in the serum of women with ovarian cancer in combination with other markers may prove useful in the future in the diagnosis of ovarian cancer, but this requires further studies.

## Figures and Tables

**Table 1:** Sensitivity and specificity of ovarian cancer detection by determination of caspase-3 and CA 125 antigen in serum of women.

Marker	Sensitivity	Specificity
Caspase-3	95%	100%
CA 125	100%	61%

**Table 2:** Sensitivity and specificity of ovarian cancer detection by determination of caspase-8 and CA 125 antigen in serum of women.

Marker	Sensitivity	Specificity
Caspase-8	100%	67%
CA 125	100%	61%

**Table 3:** Sensitivity and specificity of ovarian cancer detection by determination of caspase-9 and CA 125 antigen in serum of women.

Marker	Sensitivity	Specificity
Caspase-9	100%	100%
CA 125	100%	61%

## Data Availability

Data sharing not applicable.
